# Effects of dipeptidyl peptidase-4 inhibitors on beta-cell function and insulin resistance in type 2 diabetes: meta-analysis of randomized controlled trials

**DOI:** 10.1038/srep44865

**Published:** 2017-03-21

**Authors:** Xiafei Lyu, Xiaolin Zhu, Bin Zhao, Liang Du, Dawei Chen, Chun Wang, Guanjian Liu, Xingwu Ran

**Affiliations:** 1 Diabetic Foot Care Center, West China Hospital, Sichuan University, Guoxue Lane No. 37, Chengdu, Sichuan, China; 2 Department of Radiology, West China Hospital, Sichuan University, Guoxue Lane No. 37, Chengdu, Sichuan, China; 3 Global Medical Affairs, Merck Sharp & Dohme China, Shanghai, China; 4 Chinese Evidence-Based Medicine Centre, Chinese Cochrane Center, West China Hospital, Sichuan University, Guoxue Lane No. 37, Chengdu, Sichuan, China; 5 Department of Endocrinology and Metabolism, West China Hospital, Sichuan University, Guoxue Lane No. 37, Chengdu, Sichuan, China

## Abstract

Dipeptidyl peptidase-4 (DPP-4) inhibitors are a novel family of glucose-lowering agents. Accumulating evidence suggests that DPP-4 inhibitors preserve pancreatic beta-cell function, but results in previous studies have been inconsistent. We assessed the effects of DPP-4 inhibitors on the homoeostasis model assessment beta-cell function (HOMA-B) or insulin resistance (HOMA-IR) index in patients with type 2 diabetes through a systematic review and meta-analysis of randomized controlled trials (RCTs). Relevant articles were identified from PubMed, Embase, and Cochrane Library databases up to December 27, 2016. We calculated weighted mean differences (WMDs) and 95% confidence intervals (CIs) in each included trial and pooled the data using a random-effects model. Fifty-two trials were included in the present analysis. Compared with placebo control, DPP-4 inhibitors as monotherapy significantly improved HOMA-B (WMD 9.15; 95% CI 7.48, 10.81). Similarly, DPP-4 inhibitors as add-on therapy in combination with other drugs showed significant improvement in HOMA-B (WMD 9.04; 95% CI 5.72, 12.37). However, we found no significant improvement in HOMA-IR following treatment with DPP-4 inhibitors as mono-therapy or as add-on therapy. In conclusion, DPP-4 inhibitors as monotherapy or as add-on therapy significantly improved beta-cell function but had no significant effect on insulin resistance in type 2 diabetes.

Type 2 diabetes mellitus (T2DM) is characterized by a progressive decline in beta-cell function with insulin resistance^1^,^2^,^3^. Beta-cell dysfunction and insulin resistance are the central mechanisms in the pathogenesis of T2DM. As a surrogate marker for measuring beta-cell function and insulin sensitivity, the homoeostasis model assessment (HOMA) indexes, which are based on fasting glucose and insulin levels, have been widely used for decades in clinical and epidemiological research^4^,^5^. The validity of HOMA indexes have been confirmed against the hyperglycemic and euglycemic clamps and the intravenous glucose tolerance test^4^. Recently, the Whitehall II study has shown that beta-cell function and insulin sensitivity as measured by HOMA beta-cell function (HOMA-B) and HOMA insulin resistance (HOMA-IR) may undergo significant reduction several years before the diagnosis of T2DM^6^. The UK Prospective Diabetes Study (UKPDS) has demonstrated that HOMA-B continues to deteriorate in association with progressively increasing hyperglycemia despite treatment^7^. These data highlight the importance of preserving beta-cell function and insulin sensitivity in the prevention and management of T2DM.

Dipeptidyl peptidase-4 (DPP-4) inhibitors are a novel family of glucose-lowering agents that are increasingly used in clinical practice in treating T2DM patients. DPP-4 is responsible for the degradation of incretin hormones such as glucagon-like peptide 1 (GLP-1)^8^. Inhibition of DPP-4 reduces glycemia, sustains insulin levels, and reduces glucagon levels in T2DM patients^9^. DPP-4 inhibitors have many favorable features including a low risk of hypoglycemia and a neutral effect on body weight^8^. In addition, they are efficacious and well tolerated as mono-therapy and also as add-on therapy in combination with commonly prescribed anti-diabetic agents and are suitable for once-daily oral dosing^8^,^10^. As a result, the American Diabetes Association and the European Association for the Study of Diabetes recommend DPP-4 inhibitors as part of a combination therapy with metformin and/or other agents or as a preferable monotherapy choice for patients in whom metformin is contraindicated or not tolerated^11^,^12^.

The underlying mechanisms of DPP-4 inhibitors in the management of T2DM remain to be understood, and the roles of DPP-4 inhibitors on beta-cell function and insulin sensitivity are of particular interest. Experimental data show that DPP-4 inhibitors may help preserve pancreatic beta-cell function^13^,^14^. However, results from previous studies in humans have been inconsistent, partly because of the limited sample size and insufficient statistical power in some individual studies. In this study, we aimed to systematically review the available evidence and quantitatively summarize the findings by performing a meta-analysis of randomized controlled trials (RCTs).

## Material and Methods

This study was conducted in accordance with the Preferred Reporting Items for Systematic Reviews and Meta-Analyses (PRISMA) statement^15^.

### Study selection

Articles were eligible for inclusion if they fulfilled all the following criteria: (i) they were RCTs; (ii) the participants were patients with T2DM; (iii) they compared a DPP-4 inhibitor as monotherapy or add-on therapy with an appropriate control; (iv) they provided information on HOMA-estimated beta-cell function or insulin resistance; (v) the study duration was no less than 12 weeks; and (vi) the results were published in peer-reviewed journal as a full paper. We excluded the following types of articles: review articles or editorials, non-human studies (i.e., cell culture or animal studies), studies that did not include DPP-4 inhibitor treatment in the intervention group, and studies that did not evaluate beta-cell function or insulin resistance. Disagreement about eligibility was resolved by consensus between all authors.

### Literature search

We performed a comprehensive literature search in the PubMed, EMBASE, and Cochrane Library databases. The last search update was conducted on up to December 27, 2016. In terms of the database search strategy, we used a combination of free text (e.g., type 2 diabetes) and subheadings from MeSH (e.g., “Diabetes Mellitus, Type 2” [Mesh]) or EMTREE terms (e.g., ‘non insulin dependent diabetes mellitus’/exp). In addition to using the generic term for DPP-4 inhibitors, we also specifically named each major DPP-4 inhibitor (e.g., Sitagliptin, Vildagliptin, Saxagliptin, Linagliptin, Anagliptin, Teneligliptin, Alogliptin, Gemigliptin, and Dutogliptin) when conducting the literature search. More detailed search terms are listed in the Supplementary Materials. The reference lists of relevant studies and review articles were also checked to identify additional relevant studies.

### Data extraction

The following data were extracted from each eligible article: the first author’s name, year of publication, sample size, participants’ age, T2DM duration, types of DPP-4 inhibitors tested, study duration, mean baseline HbA1c, and mean and standard deviation of HOMA indexes in the comparison groups. When necessary, we contacted the corresponding authors of the original articles by email to request relevant data or information. If multiple articles were published using data from the same study, we extracted the report with the information most relevant to the analysis. If the same trial reported data at different follow-up durations^16^,^17^,^18^, we extracted the data corresponding to the longest follow-up period^18^. If a study reported results on the effects of DPP-4 inhibitors at different doses, we extracted data corresponding to the standard dosages for each DPP-4 inhibitor (e.g., sitagliptin 100 mg/day; saxagliptin 5 mg/day; vildagliptin 100 mg/day; linagliptin 5 mg/day; teneligliptin 20 mg/day; and alogliptin 25 mg/day) unless otherwise specified.

### Quality assessment of the included RCTs

Assessment of risk of bias in the included RCTs was performed according to the Cochrane Collaboration’s tool^19^, which includes the following domains: random sequence generation, allocation concealment, blinding of participants and personnel, blinding of outcome assessment, incomplete outcome data, selective reporting, and other bias. The risk of bias in each domain was assessed as low, high, or unclear.

### Data synthesis and statistical analysis

We calculated weighted mean differences (WMDs) and 95% confidence intervals (CIs) for the change in HOMA indexes from baseline to the end of the trial for DPP-4 inhibitors versus controls reported in each of the included trials and pooled the data in the meta-analysis using a random-effects model^20^. If both adjusted (typically derived from analysis of covariance) and unadjusted changes from baseline were reported in a trial, we selected the adjusted estimates as they accounted for possible baseline imbalance and the correlation between baseline and follow-up measures. When the adjusted estimates were not available, we limited meta-analyses to those trials with baseline balance in HOMA indexes^21^. When standard deviations of the change from baseline were not reported in the article, we converted standard errors or 95% CIs to standard deviations.

We used forest plots to visualize the effect sizes in each individual study and the pooled overall effect size. We assessed between-study heterogeneity using the χ^2^-based Cochrane’s Q statistic and the *I*^2^ metric (*I*^2^ values of 25%, 50%, and 75% were considered to indicate low, medium, and high heterogeneity, respectively)^22^. Potential of publication bias is assessed by funnel plots, in which asymmetrical plot indicates presence of reporting bias. To explore the possible differences in the effectiveness of individual DPP-4 inhibitors on beta-cell function and insulin sensitivity, we performed stratification analysis according to the type of DPP-4 inhibitor tested. Sensitivity analyses were performed by omitting one study at a time and computing the pooled effect size of the remaining studies to evaluate whether the results were affected markedly by a single study. All statistical analyses were performed using Stata software version 11.0 (Stata Corp, College Station, TX, USA).

## Results

### Characteristics of the included studies

**Figure 1:**
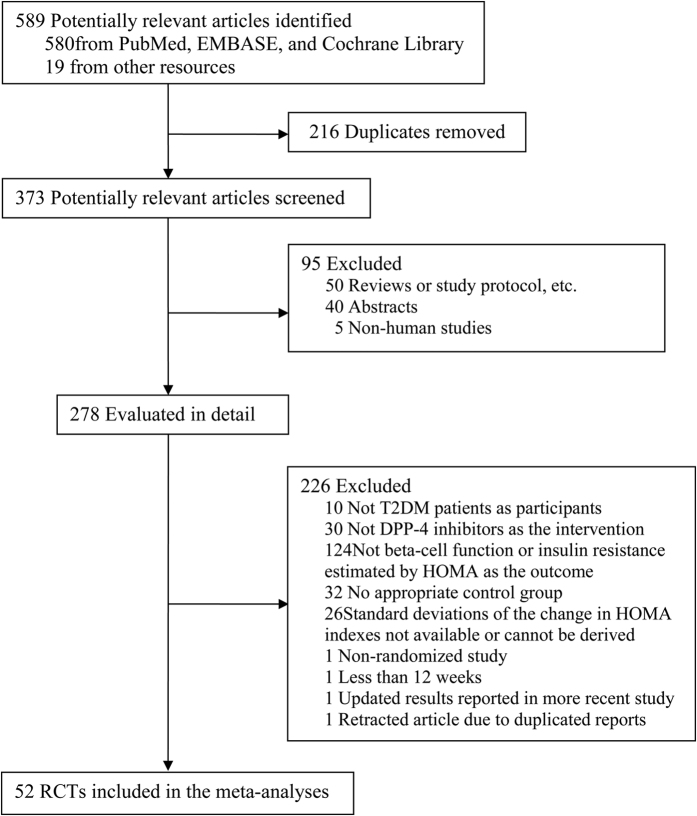
Flow chart of literature search and study selection.

We identified 589 potentially relevant articles from database search and other resources. After screening, 278 articles were evaluated in detail and 52 articles presenting RCTs^16^,^18^,^23^,^24^,^25^,^26^,^27^,^28^,^29^,^30^,^31^,^32^,^33^,^34^,^35^,^36^,^37^,^38^,^39^,^40^,^41^,^42^,^43^,^44^,^45^,^46^,^47^,^48^,^49^,^50^,^51^,^52^,^53^,^54^,^55^,^56^,^57^,^58^,^59^,^60^,^61^,^62^,^63^,^64^,^65^,^66^,^67^,^68^,^69^,^70^,^71^,^72^ were finally included in the meta-analysis (Fig. 1). Of them, 23 articles reported data from trials using DPP-4 inhibitors as monotherapy, 28 articles from trials using DPP-4 inhibitors as adds-on therapy, and the other one article involving mixed study design using DPP-4 inhibitors as both monotherapy and adds-on therapy.

The baseline characteristics of the included RCTs in the meta-analysis are shown in Table 1. The majority of studies were conducted in Caucasian or Asian populations. Sitagliptin was the most investigated drug among DPP-4 inhibitors as monotherapy and add-on therapy in the included studies. Most of the included RCTs had a relatively short study duration (typically 12 or 24 weeks). The risk of bias varied across the individual trials. All the included trials, except one^56^, involved appropriate double blind procedures, and none were associated with concerns about selective outcome reporting. However, in some of the included trials, HOMA-IR or HOMA-B data were missing for more than 20% of the participants, because these indicators were not the primary outcome and therefore were not assessed all participants. In addition, information regarding random generation sequence, allocation concealment, and blinding of outcome assessment was not clearly described in most trials (Supplementary Table 1).

### Effects of DPP-4 inhibitors as monotherapy on beta-cell function and insulin resistance

**Figure 2:**
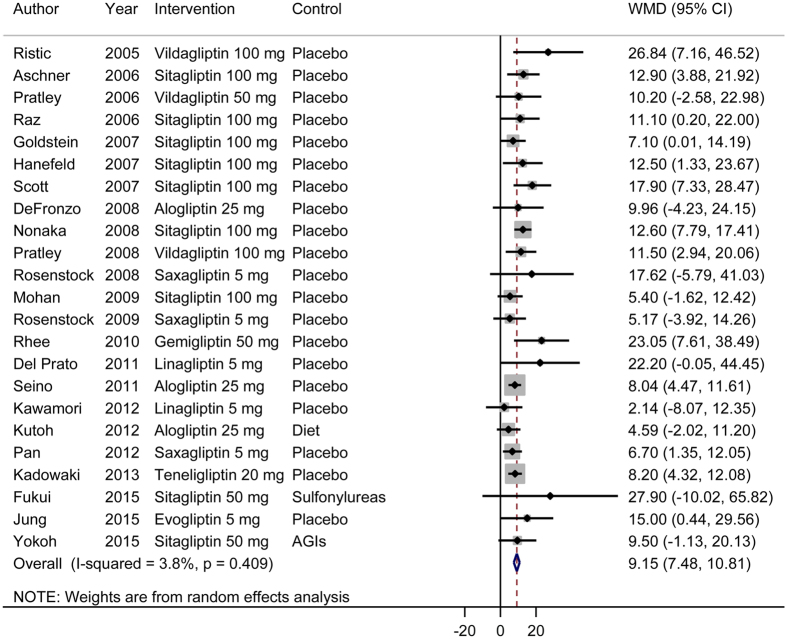
Effects of DPP-4 inhibitors as monotherapy (DPP-4 inhibitors versus placebo) on beta-cell function. WMD, weighted mean difference; CI, confidence interval.

**Figure 3:**
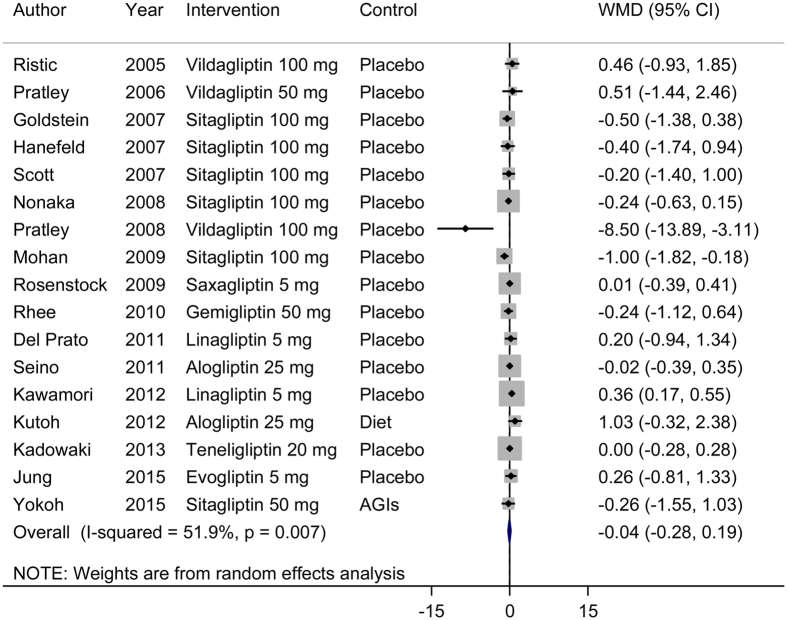
Effects of DPP-4 inhibitors (monotherapy versus placebo) on insulin resistance. WMD, weighted mean difference; CI, confidence interval.

Our meta-analysis of 23 RCTs showed that DPP-4 inhibitors as monotherapy significantly improved beta-cell function (Fig. 2). The pooled WMD (95% CI) for the HOMA-B was 9.15 (7.48, 10.81). We observed no evidence for significant heterogeneity (*I*^2^ = 4%, *P* = 0.41). For insulin resistance, we found no significant improvement after treatment with DPP-4 inhibitors as monotherapy (Fig. 3). Sensitivity analyses conducted by omitting one study at a time did not show significant alteration of the results.

### Effects of DPP-4 inhibitors as add-on therapy on beta-cell function and insulin resistance

**Figure 4:**
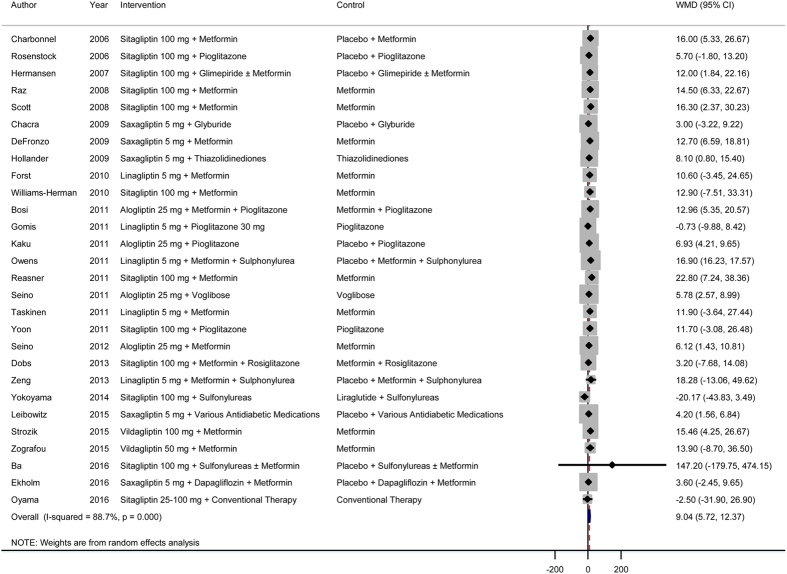
Effects of DPP-4 inhibitors as add-on therapy (DPP-4 inhibitors + other drugs versus placebo + the same other drugs) on beta-cell function. WMD, weighted mean difference; CI, confidence interval.

**Figure 5:**
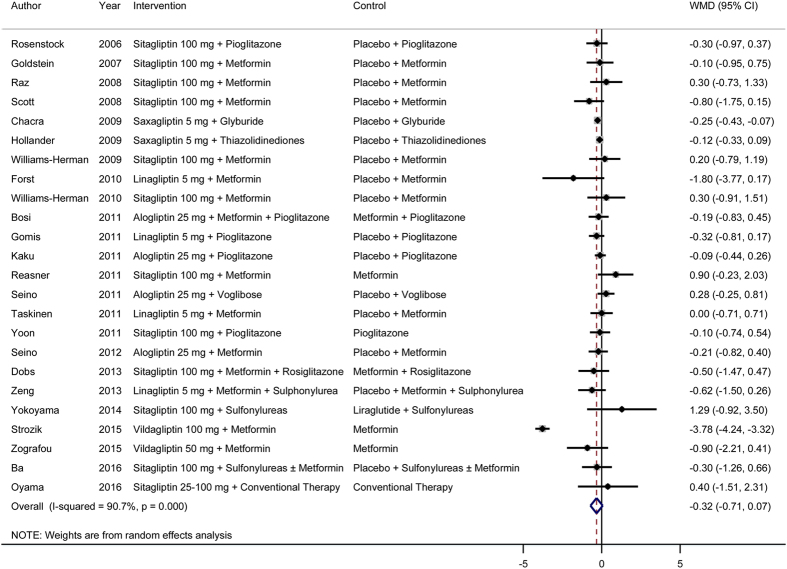
Effects of DPP-4 inhibitors as add-on therapy (DPP-4 inhibitors + other drugs versus placebo + the same other drugs) on insulin resistance. WMD, weighted mean difference; CI, confidence interval.

In a meta-analysis of 28 RCTs, we found that treatment with DPP-4 inhibitors as add-on therapy in combination with other drugs significantly improved beta-cell function (Fig. 4). The pooled WMD (95% CI) for the HOMA-B was 9.04 (5.72, 12.37). There was evidence for significant heterogeneity (*I*^2^ = 89%, *P* < 0.001). Similar to the findings for DPP-4 inhibitor monotherapy, we found no significant improvement in insulin resistance after treatment with DPP-4 inhibitors as monotherapy (Fig. 5). Sensitivity analyses conducted by omitting one study at a time did not show significant alteration of the results.

### Publication bias

In the funnel plots, we observed evidence of publication bias (i.e., asymmetrical plots) in the meta-analysis of DPP-4 inhibitors as either monotherapy (Supplementary Figures 1 and 2) or add-on therapy (Supplementary Figures 3 and 4) on beta-cell function and insulin resistance. However, these results should be interpreted with caution, because asymmetrical plots may also be a result of other reasons, such as small study effects, rather than publication bias.

### Comparative effects of individual DPP-4 inhibitors as monotherapy

In stratification analyses according to the types of DPP-4 inhibitors, we pooled data for each DPP-4 inhibitor. We found that treatment with all types of DDP-4 inhibitors except for linagliptin resulted in an increase in HOMA-B (Supplementary Figure 5). In the meta-analysis of the effects of individual DPP-4 inhibitors on insulin resistance (Supplementary Figure 6), we observed a significant improvement in insulin resistance with sitagliptin treatment without evidence of significant heterogeneity (WMD −0.38; 95% CI −0.69, −0.08; *I*^2^ = 0%, *P* for heterogeneity = 0.58). Linagliptin seemed to have a trend to increase insulin resistance, but the results should be interpreted cautiously due to the limited number of available studies. We found no significant effects of other DPP-4 inhibitors on insulin resistance.

## Discussion

Our meta-analysis of data from 52 RCTs showed that DPP-4 inhibitors as monotherapy or as add-on therapy significantly improved beta-cell function as measured by the HOMA-B. However, there was no significant improvement in insulin resistance with the use of DPP-4 inhibitors as monotherapy or as add-on therapy in combination with other drugs. Consistent with the results of previous meta-analyses^73^,^74^, these findings provide important insight into the pathophysiological mechanisms of DPP-4 inhibitor action in the treatment of T2DM.

Our results regarding the effects of DPP-4 inhibitor treatment on beta-cell function were largely consistent with results from the largest trials using DPP-4 inhibitors as monotherapy^75^, adds-on therapy^66^, or mixed trial design involving multiple trials^62^. In the trial by Pratley *et al*.^75^, vildagliptin monotherapy yielded consistently robust improvement in beta-cell function, measured not only by HOMA-B based on fasting glucose and insulin but also by meal test-derived measures, across a broad spectrum of drug-naive patients with T2DM. In addition, our findings were consistent with those of other studies that measured dynamic beta-cell function after meal ingestion. For instance, in a meal tolerance test performed with a double tracer technique (3-(3)H-glucose iv and 1-(14)C-glucose orally), the DPP-4 inhibitor vildagliptin significantly increased the insulin secretion rate divided by plasma glucose by 29% in T2DM patients^76^. When added to metformin therapy in patients with T2DM, vildagliptin treatment resulted in a significant increase in insulin secretion (postmeal suprabasal area under the 0- to 30-min C-peptide curve divided by the 30-min increase in glucose)^77^.

The favorable effects of DPP-4 inhibitors for improving beta-cell function among patients with T2DM are biologically plausible^78^. DPP-4 inhibitor treatment in diabetic animal models stimulated beta-cell survival, facilitated islet neogenesis, enhanced insulin biosynthesis^79^, and preserved beta-cell mass and function^13^. Moreover, DPP-4 gene knockout mice showed better insulin sensitivity and less pancreatic islet hypertrophy and were resistant to streptozotocin-induced loss of beta-cell mass and hyperglycemia^80^. In humans with T2DM, DPP-4 inhibitors have demonstrated improvement of beta-cell function both in the fasting and postprandial statuses, and these beneficial effects were sustained in studies with a duration up to 2 years^78^. DPP-4 inhibitors may restore beta-cell function and survival in isolated human islets through glucagon-like peptide (GLP)-1 stabilization^81^. In addition, DPP-4 inhibitors also exert an anti-inflammatory effect^82^,^83^, which may alleviate the loss of beta-cell function^84^. Recently, it is reported that DPP-4 inhibitors may protect pancreatic beta-cells by elongating the telomere length^85^. However, there is a lack of evidence in humans to demonstrate the durable effects of DPP-4 inhibitors on beta-cell function after cessation of therapy in patients with T2DM, which warrants further investigation in long-term, well-designed trials with a sufficiently long washout period after discontinuation of the study drug^78^.

In general, DPP-4 inhibitors showed no effects on insulin resistance. While the underlying mechanisms remain to be elucidated, this finding may be at least partly due to the neutral effects of DPP-4 inhibitors on body weight^8^. Interestingly, our meta-analysis showed that sitagliptin, but not the other tested DPP-4 inhibitors, as monotherapy resulted in significant improvement in insulin resistance. This finding needs to be confirmed in future studies.

The study has strengths in that it was conducted using a systematic approach and the results are based on a large number of published RCTs. However, there are several limitations. First, most of the included studies had a follow-up duration of less than 6 months. The longer-term effects of DPP-4 inhibitor treatment on beta-cell function and insulin resistance in patients with T2DM warrant further investigation. Second, our study had a lack of power to compare the effectiveness of DPP-4 inhibitors in this study, because the number of available trials for some individual DPP-4 inhibitors was still limited. DPP-4 inhibitors have similar but still varying pharmacological features^86^,^87^, and thus, their effects on beta-cell function and insulin resistance may vary. Third, we used the HOMA-B as the indicator of beta-cell function. Because this parameter is originally estimated based on fasting glucose and insulin levels, it may not sufficiently represent postprandial beta-cell function. Fourth, publication bias may exist regarding the effects of DPP-4 inhibitors on beta-cell function.

In conclusion, among patients with T2DM, DPP-4 inhibitors as monotherapy or as add-on therapy in combination with other drugs significantly improved beta-cell function but had no significant effect on insulin resistance.

## Additional Information

**How to cite this article**: Lyu, X. *et al*. Effects of dipeptidyl peptidase-4 inhibitors on beta-cell function and insulin resistance in type 2 diabetes: meta-analysis of randomized controlled trials. *Sci. Rep.* **7**, 44865; doi: 10.1038/srep44865 (2017).

**Publisher's note:** Springer Nature remains neutral with regard to jurisdictional claims in published maps and institutional affiliations.

## Supplementary Material

Supplementary Materials

## Figures and Tables

**Table 1:** Characteristics of the RCTs included in the meta-analysis.

Author, year	Study design	Intervention	Control	No. of subjects (Int/Ctrl)	Mean age (years)	Mean BMI (kg/m^2^)	Mean T2DM duration (years)	Mean baseline HbA1c	Study duration (weeks)
Ristic *et al*.^23^	Monotherapy	Vildagliptin 100 mg	Placebo	53/58	55.0	31.4	2.7	7.7	12
Aschner *et al*.^24^	Monotherapy	Sitagliptin 100 mg	Placebo	238/253	54.0	30.5	4.5	8.0	24
Charbonnel *et al*.^25^	Add-ons	Sitagliptin 100 mg + Metformin	Placebo + Metformin	464/237	54.5	31.0	6.3	8.0	24
Pratley *et al*.^26^	Monotherapy	Vildagliptin 50 mg	Placebo	70/28	54.0	30.0	4.0	8.1	12
Raz *et al*.^27^	Monotherapy	Sitagliptin 100 mg	Placebo	205/110	55.5	32.0	4.6	8.0	18
Rosenstock *et al*.^28^	Add-ons	Sitagliptin 100 mg + Pioglitazone	Placebo + Pioglitazone	175/178	56.0	31.5	6.1	8.0	24
Goldstein *et al*.^16^	Monotherapy	Sitagliptin 100 mg	Placebo	179/176	53.4	32.0	4.5	8.8	24
Hanefeld *et al*.^29^	Monotherapy	Sitagliptin 100 mg	Placebo	110/111	56.0	31.5	3.5	7.7	12
Hermansen *et al*.^30^	Add-ons	Sitagliptin 100 mg + Glimepiride ± Metformin	Placebo + Glimepiride ± Metformin	222/219	55.0	31.0	8.8	8.3	24
Scott *et al*.^31^	Monotherapy	Sitagliptin 100 mg	Placebo	124/125	55.2	31.0	4.5	9.5	12
DeFronzo *et al*.^32^	Monotherapy	Alogliptin 25 mg	Placebo	131/64	53.4	NR	NR	7.9	26
Nonaka *et al*.^33^	Monotherapy	Sitagliptin 100 mg	Placebo	75/76	55.3	25.0	4.0	7.6	12
Pratley *et al*.^34^	Monotherapy	Vildagliptin 100 mg	Placebo	1470/182	53.0	32.3	2.2	8.5	24
Raz *et al*.^35^	Add-ons	Sitagliptin 100 mg + Metformin	Metformin	96/94	54.0	30.3	7.8	9.2	18
Rosenstock *et al*.^36^	Monotherapy	Saxagliptin 5 mg	Placebo	106/95	54.0	31.0	2.4	8.0	12
Scott *et al*.^37^	Add-ons	Sitagliptin 100 mg + Metformin	Metformin	94/92	55.3	30.0	5.2	7.8	18
Chacra *et al*.^38^	Add-ons	Saxagliptin 5 mg + Glyburide	Placebo + Glyburide	253/267	55.0	29.0	6.8	8.4	24
DeFronzo *et al*.^39^	Add-ons	Saxagliptin 5 mg + Metformin	Metformin	191/179	54.8	31.4	6.5	8.1	24
Hollander *et al*.^40^	Add-ons	Saxagliptin 5 mg + Thiazolidinediones	Thiazolidinediones	186/184	53.6	30.0	5.2	8.3	24
Mohan *et al*.^41^	Monotherapy	Sitagliptin 100 mg	Placebo	352/178	50.0	25.0	2.0	8.8	18
Rosenstock *et al*.^42^	Monotherapy	Saxagliptin 5 mg	Placebo	106/95	54.0	31.0	2.4	8.0	24
Forst *et al*.^43^	Add-ons	Linagliptin 5 mg + Metformin	Metformin	66/71	60.0	32.0	6.8	8.5	12
Rhee *et al*.^44^	Monotherapy	Gemigliptin 50 mg	Placebo	35/34	52.0	25.3	4.4	8.2	12
Williams-Herman *et al*.^18^	Add-ons	Sitagliptin 100 mg + Metformin	Metformin	107/88	54.0	31.5	4.2	8.7	104
Bosi *et al*.^45^	Add-ons	Alogliptin 25 mg + Metformin + Pioglitazone	Metformin + Pioglitazone	404/399	55.0	31.5	7.0	8.1	52
Del Prato *et al*.^46^	Monotherapy	Linagliptin 5 mg	Placebo	336/167	55.0	29.1	NR	8.0	24
Gomis *et al*.^47^	Add-ons	Linagliptin 5 mg + Pioglitazone	Pioglitazone	259/130	57.4	29.0	NR	8.6	24
Kaku *et al*.^48^	Add-ons	Alogliptin 25 mg + Pioglitazone	Placebo + Pioglitazone	113/115	60.0	26.3	6.8	7.9	12
Owens *et al*.^49^	Add-ons	Linagliptin 5 mg + Metformin + Sulphonylurea	Placebo + Metformin + Sulphonylurea	541/175	58.0	28.0	NR	8.1	24
Reasner *et al*.^50^	Add-ons	Sitagliptin 100 mg + Metformin	Metformin	625/621	50.0	33.0	3.4	9.9	18
Seino *et al*.^51^	Add-ons	Alogliptin 25 mg + Voglibose	Voglibose	79/75	62.5	24.0	8.0	8.0	12
Seino *et al*.^52^	Monotherapy	Alogliptin 25 mg	Placebo	80/75	59.3	24.5	6.9	7.9	12
Taskinen *et al*.^53^	Add-ons	Linagliptin 5 mg + Metformin	Metformin	523/177	56.6	30.0	NR	8.1	24
Yoon *et al*.^54^	Add-ons	Sitagliptin 100 mg + Pioglitazone	Pioglitazone	261/259	51.0	29.7	2.4	9.5	24
Kawamori *et al*.^55^	Monotherapy	Linagliptin 5 mg	Placebo	159/80	60.0	24.5	NR	8.0	12
Kutoh *et al*.^56^	Monotherapy	Alogliptin 25 mg	Diet	25/26	48.0	26.3	0	10.3	12
Pan *et al*.^57^	Monotherapy	Saxagliptin 5 mg	Placebo	284/284	51.4	25.9	1.0	8.1	24
Seino *et al*.^58^	Add-ons	Alogliptin 25 mg + Metformin	Metformin	96/100	52.2	25.0	6.3	8.0	12
Dobs *et al*.^59^	Add-ons	Sitagliptin 100 mg + Metformin + rosiglitazone	Metformin + rosiglitazone	170/92	54.6	30.5	9.3	8.8	54
Kadowaki *et al*.^60^	Monotherapy	Teneligliptin 20 mg	Placebo	79/80	58.8	25.1	6.0	7.9	12
Zeng *et al*.^61^	Add-ons	Linagliptin 5 mg + Metformin + Sulphonylurea	Placebo + Metformin + Sulphonylurea	144/48	56.0	25.8	NR	8.1	12
Heise *et al*.^62^	Mixed	Linagliptin 5 mg + Various antidiabetic medications	Placebo + Various antidiabetic medications	1905/796	57.0	29.3	NR	8.2	24
Yokoyama *et al*.^63^	Add-ons	Sitagliptin 100 mg + Sulfonylureas	Liraglutide + Sulfonylureas	49/50	61.3	25.9	11.3	7.8	24
Fukui *et al*.^64^	Monotherapy	Sitagliptin 50 mg	Sulfonylureas	21/22	66.3	26.3	7.5	7.3	24
Jung *et al*.^65^	Monotherapy	Evogliptin (DA1229) 5 mg	Placebo	43/48	54.3	25.5	3.7	7.6	12
Leibowitz *et al*.^66^	Add-ons	Saxagliptin 5 mg + Various antidiabetic medications	Placebo + Various antidiabetic medications	2408/2312	65.2	31.1	8.3	7.5	151
Strozik *et al*.^67^	Add-ons	Vildagliptin 100 mg + Metformin	Metformin	17/16	55.0	32.0	NR	8.1	12
Yokoh *et al*.^68^	Monotherapy	Sitagliptin 50 mg	Alpha-glucosidase inhibitor	58/58	58.5	26.1	6.8	7.6	24
Zografou *et al*.^69^	Add-ons	Vildagliptin 50 mg + Metformin	Metformin	32/32	54.4	31.9	NR	8.1	24
Ba *et al*.^70^	Add-ons	Sitagliptin 100 mg + Sulfonylureas ± Metformin	Placebo + Sulfonylureas ± Metformin	249/249	56.0	25.4	7.0	8.5	24
Ekholm *et al*.^71^	Add-ons	Saxagliptin 5 mg + Dapagliflozin + Metformin	Placebo + Dapagliflozin + Metformin	160/152	54.0	32	7.3	8.9	24
Oyama *et al*.^72^	Add-ons	Sitagliptin 25–100 mg + Conventional therapy	Conventional therapy	222/220	69.3	25.1	NR	7.0	24

Abbreviations: Int, intervention group; Ctrl, control group; BMI, body mass index; T2DM, type 2 diabetes mellitus; HbA1c, hemoglobin A1c; NR, not reported.
