# Supplementary material for: Effects of dipeptidyl peptidase-4 inhibitors on beta-cell function and insulin resistance in type 2 diabetes: meta-analysis of randomized controlled trials
Source: Sci Rep. 2017 Mar 21;7:44865. doi: 10.1038/srep44865 (PMC5359588; doi:10.1038/srep44865)
Supplement: Supplementary Materials [file srep44865-s1.doc]

**Effects of dipeptidyl peptidase-4 inhibitors on beta-cell function and insulin resistance in type 2 diabetes: meta-analysis of randomized controlled trials**

**Short title: DPP-4 inhibitors effect in type 2 diabetes**

**Xiafei Lyu^1,5^, Xiaolin Zhu^3^, Bin Zhao^3^, Liang Du^4^, Dawei Chen^1,2^, Chun Wang^1,2^, Guanjian Liu^4^, Xingwu Ran^1,2*^**

^1^ Diabetic Foot Care Center, West China Hospital, Sichuan University, Guoxue Lane No. 37, Chengdu, Sichuan, China.

^2^ Department of Endocrinology and Metabolism, West China Hospital, Sichuan University, Guoxue Lane No. 37, Chengdu, Sichuan, China.

^3^Global Medical Affairs, Merck Sharp & Dohme China, Shanghai, China,

^4^ Chinese Evidence-Based Medicine Centre, Chinese Cochrane Center, West China Hospital, Sichuan University, Guoxue Lane No. 37, Chengdu, Sichuan, China.

^5^ Department of Radiology, West China Hospital, Sichuan University, Guoxue Lane No. 37, Chengdu, Sichuan, China.

*Corresponding author: Xingwu Ran, Diabetic Foot Care Center, Department of Endocrinology and Metabolism, West China Hospital, Sichuan University, Guoxue Lane No. 37, Chengdu, Sichuan 610041, China

Email: ranxingwu@sina.com; [ranxingwu@163.com](mailto:ranxingwu@163.com); Tel: +8618980601305

**Supplementary Text**

**Search terms**

**PubMed:**

("Dipeptidyl-Peptidase IV Inhibitors"[Mesh] or Dipeptidyl peptidase-4 inhibitor or DPP-4 inhibitor or DPP4 inhibitor or Sitagliptin or Vildagliptin or Saxagliptin or Linagliptin or Anagliptin or Teneligliptin or Alogliptin or Gemigliptin or Dutogliptin) and (β-cell or beta-cell function or insulin secretion or insulin sensitivity or insulin resistance or homeostatic model assessment or HOMA) and ("Diabetes Mellitus, Type 2"[Mesh] or type 2 diabetes or type 2 diabetic) and (trial or randomiz* or placebo) not (review[pt] or editorial[pt] or comment[pt])

**EMBASE:**

('dipeptidyl peptidase IV inhibitor'/exp or dipeptidyl peptidase-4 inhibitor or DPP-4 inhibitor or DPP4 inhibitor or Sitagliptin or Vildagliptin or Saxagliptin or Linagliptin or Anagliptin or Teneligliptin or Alogliptin or Gemigliptin or Dutogliptin) and (beta-cell function or insulin secretion or insulin sensitivity or insulin resistance or homeostatic model assessment or HOMA) and ('non insulin dependent diabetes mellitus'/exp OR 'type 2 diabetes':ab,ti OR 'type 2 diabetic':ab,ti OR 'non-insulin dependent diabetes':ab,ti OR 'non-insulin dependent diabetic':ab,ti) and ([controlled clinical trial]/lim or [randomized controlled trial]/lim) and [humans]/lim and ([embase]/lim or [embase classic]/lim) and ([article]/lim or [article in press]/lim)

**Cochrane Library:**

(Dipeptidyl peptidase-4 inhibitor or DPP-4 inhibitor or DPP4 inhibitor or Sitagliptin or Vildagliptin or Saxagliptin or Linagliptin or Anagliptin or Teneligliptin or Alogliptin or Gemigliptin or Dutogliptin) and (beta-cell function or insulin secretion or insulin sensitivity or insulin resistance or homeostatic model assessment or HOMA) and (type 2 diabetes or type 2 diabetic)

**Supplementary Figure 1. Funnel plots of DPP-4 inhibitors as monotherapy (DPP-4 inhibitors versus placebo) on beta-cell function.**

**Supplementary Figure 2. Funnel plots of DPP-4 inhibitors as monotherapy (DPP-4 inhibitors versus placebo) on insulin resistance.**

**Supplementary Figure 3. Funnel plots of DPP-4 inhibitors as add-ons therapy (DPP-4 inhibitors + other drugs versus placebo + the same other drugs) on beta-cell function.**

**Supplementary Figure 4. Funnel plots of DPP-4 inhibitors as add-ons therapy (DPP-4 inhibitors + other drugs versus placebo + the same other drugs) on insulin resistance.**

**Supplementary Figure 5. Effects of individual DPP-4 inhibitor as monotherapy (DPP-4 inhibitors versus placebo) on beta-cell function.**

**Supplementary Figure 6. Effects of individual DPP-4 inhibitor as monotherapy (DPP-4 inhibitors versus placebo) on insulin resistance.**

Supplementary Table 1. Assessment of risk of bias in the included trials according to the Cochrane Collaboration’s tool

| **Author, year** | **Random sequence generation** | **Allocation concealment** | **Blinding of participants and personnel** | **Blinding of outcome assessment** | **Incomplete outcome data** | **Selective reporting** | **Other bias** |
| --- | --- | --- | --- | --- | --- | --- | --- |
| Ristic et al, 2005[^23^](#_ENREF_23) | Low risk | Unclear risk | Low risk | Unclear risk | High risk | Low risk | Low risk |
| Aschner et al, 2006[^24^](#_ENREF_24) | Low risk | Unclear risk | Low risk | High risk | Low risk | Low risk | Low risk |
| Charbonnel et al, 2006[^25^](#_ENREF_25) | Low risk | Unclear risk | Low risk | High risk | Low risk | Low risk | Unclear risk |
| Pratley et al, 2006[^26^](#_ENREF_26) | Low risk | Unclear risk | Low risk | Unclear risk | Low risk | Low risk | Low risk |
| Raz et al, 2006[^27^](#_ENREF_27) | Low risk | Unclear risk | Low risk | Unclear risk | High risk | Low risk | Unclear risk |
| Rosenstock et al, 2006[^28^](#_ENREF_28) | Low risk | Unclear risk | Low risk | High risk | High risk | Low risk | Low risk |
| Goldstein et al, 2007[^16^](#_ENREF_16) | Low risk | Unclear risk | Low risk | High risk | Low risk | Low risk | Unclear risk |
| Hanefeld et al, 2007[^29^](#_ENREF_29) | Low risk | Unclear risk | Low risk | High risk | Low risk | Low risk | Low risk |
| Hermansen et al, 2007[^30^](#_ENREF_30) | Low risk | Low risk | Low risk | High risk | Low risk | Low risk | Low risk |
| Scott et al, 2007[^31^](#_ENREF_31) | Low risk | Unclear risk | Low risk | High risk | Low risk | Low risk | Low risk |
| DeFronzo et al, 2008[^32^](#_ENREF_32) | Low risk | Unclear risk | Low risk | Unclear risk | Low risk | Low risk | Unclear risk |
| Nonaka et al, 2008[^33^](#_ENREF_33) | Low risk | Unclear risk | Low risk | High risk | Low risk | Low risk | Low risk |
| Pratley et al, 2008[^34^](#_ENREF_34) | Low risk | Unclear risk | Low risk | Unclear risk | Low risk | Low risk | Low risk |
| Raz et al, 2008[^35^](#_ENREF_35) | Low risk | Unclear risk | Low risk | Unclear risk | High risk | Low risk | Low risk |
| Rosenstock et al, 2008[^36^](#_ENREF_36) | Low risk | Unclear risk | Low risk | Unclear risk | High risk | Low risk | Low risk |
| Scott et al, 2008[^37^](#_ENREF_37) | Low risk | Unclear risk | Low risk | High risk | Low risk | Low risk | Low risk |
| Chacra et al, 2009[^38^](#_ENREF_38) | Low risk | Low risk | Low risk | Low risk | Low risk | Low risk | Low risk |
| DeFronzo et al, 2009[^39^](#_ENREF_39) | Low risk | Low risk | Low risk | Unclear risk | Low risk | Low risk | Low risk |
| Hollander et al, 2009[^40^](#_ENREF_40) | Low risk | Low risk | Low risk | Unclear risk | Low risk | Low risk | Low risk |
| Mohan et al, 2009[^41^](#_ENREF_41) | Low risk | Unclear risk | Low risk | Unclear risk | Low risk | Low risk | Low risk |
| Rosenstock et al, 2009[^42^](#_ENREF_42) | Low risk | Unclear risk | Low risk | Unclear risk | Low risk | Low risk | Low risk |
| Forst et al, 2010[^43^](#_ENREF_43) | Low risk | Low risk | Low risk | Unclear risk | High risk | Low risk | Low risk |
| Rhee et al, 2010[^44^](#_ENREF_44) | Low risk | Unclear risk | Low risk | Unclear risk | Low risk | Low risk | Low risk |
| WilliamsHigh riskHerman et al, 2010[^18^](#_ENREF_18) | Low risk | Low risk | Low risk | High risk | High risk | Low risk | Low risk |
| Bosi et al, 2011[^45^](#_ENREF_45) | Low risk | Unclear risk | Low risk | Unclear risk | Low risk | Low risk | Low risk |
| Del Prato et al, 2011[^46^](#_ENREF_46) | Low risk | Low risk | Low risk | Unclear risk | Low risk | Low risk | Low risk |
| Gomis et al, 2011[^47^](#_ENREF_47) | Low risk | Unclear risk | Low risk | Unclear risk | Low risk | Low risk | Low risk |
| Kaku et al, 2011[^48^](#_ENREF_48) | Low risk | Low risk | Low risk | High risk | High risk | Low risk | Low risk |
| Owens et al, 2011[^49^](#_ENREF_49) | Low risk | Unclear risk | Low risk | Unclear risk | Low risk | Low risk | Low risk |
| Reasner et al, 2011[^50^](#_ENREF_50) | Low risk | Unclear risk | Low risk | Unclear risk | Low risk | Low risk | Low risk |
| Seino et al, 2011[^51^](#_ENREF_51) | Low risk | Unclear risk | Low risk | Unclear risk |  | Low risk | Low risk |
| Seino et al, 2011[^52^](#_ENREF_52) | Low risk | Low risk | Low risk | High risk | Low risk | Low risk | Low risk |
| Taskinen et al, 2011[^53^](#_ENREF_53) | Low risk | Unclear risk | Low risk | Unclear risk | High risk | Low risk | Low risk |
| Yoon et al, 2011[^54^](#_ENREF_54) | Low risk | Unclear risk | Low risk | High risk | Low risk | Low risk | Low risk |
| Kawamori et al, 2012[^55^](#_ENREF_55) | Low risk | Unclear risk | Low risk | Unclear risk | High risk | Low risk | Low risk |
| Kutoh et al, 2012[^56^](#_ENREF_56) | Low risk | Unclear risk | High risk | Unclear risk | Low risk | Low risk | Low risk |
| Pan et al, 2012[^57^](#_ENREF_57) | Low risk | Low risk | Low risk | Unclear risk | High risk | Low risk | Low risk |
| Seino et al, 2012[^58^](#_ENREF_58) | Low risk | Low risk | Low risk | Unclear risk | Low risk | Low risk | Low risk |
| Dobs et al, 2013[^59^](#_ENREF_59) | Low risk | Unclear risk | Low risk | High risk | Low risk | Low risk | Low risk |
| Kadowaki et al, 2013[^60^](#_ENREF_60) | Low risk | Unclear risk | Low risk | Low risk | Low risk | Low risk | Low risk |
| Zeng et al, 2013[^61^](#_ENREF_61) | Low risk | Unclear risk | Low risk | Unclear risk | High risk | Low risk | Low risk |
| Heise et al, 2014[^62^](#_ENREF_62) | Low risk | Unclear risk | Unclear risk | Unclear risk | High risk | Low risk | Unclear risk |
| Yokoyama et al, 2014[^63^](#_ENREF_63) | Low risk | Unclear risk | High risk | Unclear risk | Low risk | Low risk | Low risk |
| Fukui et al, 2015 [^64^](#_ENREF_64) | Low risk | Unclear risk | High risk | Unclear risk | Low risk | Low risk | Low risk |
| Jung et al, 2015[^65^](#_ENREF_65) | Low risk | Unclear risk | Low risk | Unclear risk | Low risk | Low risk | Low risk |
| Leibowitz et al, 2015 [^66^](#_ENREF_66) | Low risk | Unclear risk | Low risk | Low risk | High risk | Low risk | Low risk |
| Strozik et al, 2015[^67^](#_ENREF_67) | Low risk | Unclear risk | Low risk | Unclear risk | Unclear risk | Low risk | High risk |
| Yokoh et al, 2015 [^68^](#_ENREF_68) | Low risk | Unclear risk | High risk | Low risk | Low risk | Low risk | Low risk |
| Zografou et al, 2015 [^69^](#_ENREF_69) | Low risk | Unclear risk | High risk | Unclear risk | Low risk | Low risk | Low risk |
| Ba et al, 2016 [^70^](#_ENREF_70) | Low risk | Low risk | Low risk | Low risk | High risk | Low risk | Low risk |
| Ekholm et al, 2016 [^71^](#_ENREF_71) | Low risk | Unclear risk | Low risk | Low risk | High risk | Low risk | Low risk |
| Oyama et al, 2016 [^72^](#_ENREF_72) | Low risk | Low risk | High risk | Low risk | Low risk | Low risk | Low risk |
